# Drug Eruptions

**Published:** 1887-09

**Authors:** 


Drug Eruptions. A clinical study of the irritant effects of Drugs upon the Skin. By Prince A. Morrow, A. M., M. D., Clinical Professor of Venereal Diseases, formerly Clinical Lecturer on Dermatology in the University of the City of New York, etc., (now editor of the *Journal of Cutaneous Diseases.*) New York, Wm. Wood & Co., 1887, illustrated by two handsome chromo-lithographs showing the eruption sometimes caused by the internal use of the Bromide of Potassium. 195 pages, cloth.

This work should be read by all physicians. It is important to discriminate between an idiopathic disease of the skin and a dermatitis produced by the administration of drugs. Yet it is sometimes difficult to do. The author has evidently studied the subject well, and the book shows much and careful research. As an item of interest to our Texas readers, and apropos to the subject of "quinine rash," which has attracted some attention in Texas lately, and of a paper on the subject read at the last meeting of the State Medical Association (by Dr. Lowry, of San Antonio) on "The Untoward Effects of Quinine," and which paper appears in the the Transactions, we will state that the author enumerates no less than six distinct forms of eruption that have been observed and recorded, as being produced by the internal administration of quinine, or other salts of the bark; to-wit, an "erythematous form," which is the most common, sometimes extends over the whole surface of the body, disappears on the suspension of the drug, and is followed by desquamation; in one case desquamation continued for three months. The "urticarial form," the "papular," and the "vesicular" forms, the "petechial" form, the "bullous" form; and the gangrenous form." Aye, there are more strange things in medicine than many of us ever dreamed of in our philosophy.

The book is printed on fine paper, in good type.

